# Identification of the PANoptosome: A Molecular Platform Triggering Pyroptosis, Apoptosis, and Necroptosis (PANoptosis)

**DOI:** 10.3389/fcimb.2020.00237

**Published:** 2020-05-29

**Authors:** Shelbi Christgen, Min Zheng, Sannula Kesavardhana, Rajendra Karki, R. K. Subbarao Malireddi, Balaji Banoth, David E. Place, Benoit Briard, Bhesh Raj Sharma, Shraddha Tuladhar, Parimal Samir, Amanda Burton, Thirumala-Devi Kanneganti

**Affiliations:** Department of Immunology, St. Jude Children's Research Hospital, Memphis, TN, United States

**Keywords:** PANoptosis, PANoptosome, NLRP3, ASC, RIPK1, RIPK3, caspase-1, caspase-8

## Abstract

Programmed cell death plays crucial roles in organismal development and host defense. Recent studies have highlighted mechanistic overlaps and extensive, multifaceted crosstalk between pyroptosis, apoptosis, and necroptosis, three programmed cell death pathways traditionally considered autonomous. The growing body of evidence, in conjunction with the identification of molecules controlling the concomitant activation of all three pathways by pathological triggers, has led to the development of the concept of PANoptosis. During PANoptosis, inflammatory cell death occurs through the collective activation of pyroptosis, apoptosis, and necroptosis, which can circumvent pathogen-mediated inhibition of individual death pathways. Many of the molecular details of this emerging pathway are unclear. Here, we describe the activation of PANoptosis by bacterial and viral triggers and report protein interactions that reveal the formation of a PANoptosome complex. Infection of macrophages with influenza A virus, vesicular stomatitis virus, *Listeria monocytogenes*, or *Salmonella enterica* serovar Typhimurium resulted in robust cell death and the hallmarks of PANoptosis activation. Combined deletion of the PANoptotic components caspase-1 (CASP1), CASP11, receptor-interacting serine/threonine-protein kinase 3 (RIPK3), and CASP8 largely protected macrophages from cell death induced by these pathogens, while deletion of individual components provided reduced or no protection. Further, molecules from the pyroptotic, apoptotic, and necroptotic cell death pathways interacted to form a single molecular complex that we have termed the PANoptosome. Overall, our study identifies pathogens capable of activating PANoptosis and the formation of a PANoptosome complex.

## Introduction

The execution of programmed cell death is a complex process required for proper organismal development and homeostasis. In addition, the activation of multiple programmed cell death pathways, including pyroptosis, apoptosis, and necroptosis, is vital for host defense against pathogenic invaders. Pyroptosis and necroptosis are characterized as lytic, immunologically active forms of cell death, while apoptosis has historically been considered immunologically silent (Green et al., 2009). Though these pathways have unique characteristics, they utilize common activation mechanisms, including homotypic interactions to form large activation complexes (Kesavardhana et al., 2020). In pyroptosis, innate immune sensors respond to pathogen-associated and damage-associated molecular patterns (PAMPs and DAMPs, respectively) to mediate the formation of multimeric signaling complexes known as inflammasomes (Martinon et al., 2002). Activation of inflammasomes leads to the recruitment of apoptosis-associated speck-like protein containing a CARD (ASC), followed by the recruitment and self-activation of the inflammatory cysteine protease caspase-1 (CASP1) (Martinon et al., 2002; Cai X. et al., 2014; Franklin et al., 2014; Hu et al., 2015). Activation of CASP1 leads to the processing of other molecules, including the executioner of pyroptosis, gasdermin D (GSDMD) (Howard et al., 1991; Thornberry et al., 1992; Martinon et al., 2002; Kayagaki et al., 2015; Shi et al., 2015). After processing, the N-terminus of GSDMD oligomerizes to form pores within the cell membrane, allowing for the release of proinflammatory cytokines (Kayagaki et al., 2015; Shi et al., 2015). On the other hand, necroptosis is primarily regulated by proteins containing a receptor-interacting protein (RIP) homotypic interaction motif (RHIM), including RIP kinase 1 (RIPK1) and RIP kinase 3 (RIPK3) (Feng et al., 2007; Degterev et al., 2008; Cho et al., 2009; He et al., 2009; Zhang et al., 2009). Activation of RIPK3 leads to the phosphorylation of mixed lineage kinase domain-like (MLKL), the executioner of necroptosis (Sun et al., 2012; Zhao et al., 2012; Cai Z. et al., 2014; Chen et al., 2014; Dondelinger et al., 2014; Wang et al., 2014). Similar to the function of GSDMD in pyroptosis, the activation of MLKL leads to self-oligomerization and the formation of pores within the plasma membrane. The release of cytokines and DAMPs by pyroptosis and necroptosis leads to the propagation of inflammatory signals. Similar to the regulation of pyroptosis by the inflammasome, activation of apoptosis is regulated through the formation of large signaling platforms. The intrinsic pathway of apoptosis is mediated by the oligomerization of apoptotic protease-activating factor (APAF) to form a CASP9-activation platform known as the apoptosome (Li et al., 1997; Zou et al., 1999), while signaling through death receptors and the formation of the death-inducing signaling complex (DISC) mediates activation of CASP8 and the extrinsic pathway of apoptosis (Yang, 2015). Activation of CASP8 or CASP9 ultimately leads to the activation of executioner caspases, including CASP3 and CASP7 (Slee et al., 1999, 2001).

Studies focused on the mechanisms regulating these cell death pathways have revealed that some molecules are capable of regulating pyroptosis, apoptosis, and necroptosis. Z-DNA-binding protein (ZBP1) was found to be crucial for the activation of all three pathways after influenza A virus (IAV) infection (Kuriakose et al., 2016; Kesavardhana et al., 2017). In addition, loss of transforming growth factor beta-activated kinase 1 (TAK1) activity through genetic deletion or pathogen-mediated inhibition leads to activation of pyroptosis, apoptosis, and necroptosis (Malireddi et al., 2018, 2020; Orning et al., 2018; Sarhan et al., 2018). These findings, among others, have led to the conceptualization of PANoptosis, a form of inflammatory cell death whereby three major pathways of programmed cell death, pyroptosis, apoptosis, and necroptosis, become activated (Kuriakose et al., 2016; Kesavardhana et al., 2017; Malireddi et al., 2018, 2019, 2020; Samir et al., 2020; Zheng et al., 2020). Though ZBP1 and TAK1 have been identified as regulators of PANoptosis, there are still many unanswered questions about the mechanistic details of this emerging pathway. The coordinated activation of these pathways through PANoptosis provides an effective backup strategy for a host to circumvent pathogenic evasion strategies. Consistent with the idea of the existence of cell death backup programs, the “guard hypothesis” that was proposed in plants is well described, whereby the blockade of an innate immune signaling pathway by a pathogen results in the activation of another pathway (Dangl and Jones, 2001; Jorgensen et al., 2017). Additional studies have demonstrated that when pyroptosis is blocked, CASP8 can utilize the assembled inflammasome machinery to induce a CASP8-dependent form of cell death that has been referred to as both apoptosis and secondary pyroptosis (Sagulenko et al., 2013; Lukens et al., 2014; Gurung et al., 2016; Mascarenhas et al., 2017; Schneider et al., 2017; Van Opdenbosch et al., 2017). These findings suggest that the activation of pyroptosis, apoptosis, and necroptosis during PANoptosis is regulated by a common master death complex. The conceptualized complex would therefore form a flexible skeleton, whereby the core components of different cell death pathways may be recruited to execute inflammatory cell death. Supporting this hypothesis, studies of the cell death induced by the loss of TAK1 have revealed physical interactions between molecules known to be involved in pyroptosis, apoptosis, and necroptosis (Malireddi et al., 2020). Here, we describe the activation of PANoptosis by the bacterial pathogens *Salmonella enterica* serovar Typhimurium and *Listeria monocytogenes* and the viral triggers IAV and vesicular stomatitis virus (VSV) and show that key molecules from pyroptosis, extrinsic apoptosis, and necroptosis are capable of interacting to form a cell death complex we term the PANoptosome.

## Materials and Methods

### Mice

*Casp1*/*11*^−/−^ (Kanneganti et al., 2006b), *Gsdmd*^−/−^ (Karki et al., 2018), *Ripk3*^−/−^ (Newton et al., 2004), *Ripk3*^−/−^*Casp8*^−/−^ (Oberst et al., 2011), and *Casp1*/*11*^−/−^*Ripk3*^−/−^*Casp8*^−/−^ (Gurung et al., 2016) mice have been described previously. *Gsdmd*^−/−^*Mlkl*^−/−^mice were generated by crossing previously described *Gsdmd*^−/−^ (Karki et al., 2018) and *Mlkl*^−/−^ (Murphy et al., 2013) single knockout mice. All mice were bred at the Animal Resources Center at St. Jude Children's Research Hospital and were backcrossed to the C57BL/6 background for at least 8 generations. Single nucleotide polymorphisms (SNPs) were sequenced to confirm the genomic background. Animal studies were conducted under protocols approved by the St. Jude Children's Research Hospital committee on the Use and Care of Animals.

### Bone Marrow-Derived Macrophages (BMDMs)

Primary BMDMs were cultivated for 6 days at 37°C in IMDM (Thermo Fisher Scientific, 11995-073) supplemented with 10% FBS (Biowest, S1620), 30% L929-conditioned medium, 1% non-essential amino acids (Thermo Fisher Scientific, 11140-050), and 1% penicillin and streptomycin (Thermo Fisher Scientific, 15070-063). BMDMs were then seeded into 12-well plates with antibiotic-free medium at a density of 1 × 10^6^ cells per well and incubated overnight before stimulation or infection.

### Viral Culture

IAV (A/Puerto Rico/8/34, H1N1 [PR8]) was generated by reverse genetics as previously described (Hoffmann et al., 2000). Virus stocks were propagated by inoculation of seed virus into the allantoic cavity of 9–11-day old embryonated chicken eggs. Viral titer was measured by plaque assay in MDCK cells. VSV was propagated in Vero cells through infection of the cells at a multiplicity of infection of 0.01. Viral titer was measured by plaque assay in Vero cells.

### Bacterial Culture

*S*. Typhimurium strain SL1344 were grown in Luria-Bertani (LB) broth (MP Biomedicals, 3002-031) under aerobic conditions at 37°C overnight from a single colony. Bacteria were then subcultured separately at a ratio of 1:10 at 37°C for 4 h in fresh LB broth to reach the log phase. *Listeria monocytogenes* strain 10403S was grown from a single colony in Brain-Heart Infusion broth under aerobic conditions at 37°C.

### Cell Stimulation/Infection

For IAV and VSV infection, BMDMs were infected at a multiplicity of infection of 20 and 1, respectively, in high glucose DMEM plain media (Sigma, D6171). After adsorption for 2 h, cells were supplemented with 10% FBS and then incubated for the indicated time. For bacterial infection, the BMDMs were infected separately with *S*. Typhimurium and *L. monocytogenes* at a multiplicity of infection of 2 for 6 h and a multiplicity of infection of 5 for 8 h, respectively. For TAK1 inhibition, BMDMs were treated with 0.1 μM 5Z-7-Oxozeaenol (TAK1i) for 1 h followed by LPS (100 ng/mL) post-treatment for the indicated times.

### Immunoblot Analysis

For caspase-1 analysis, BMDMs were lysed along with the supernatant using 50 μL caspase lysis buffer (1 × protease inhibitors (Roche), 1 × phosphatase inhibitors (Roche), 10% NP-40 and 25 mM DTT) followed by the addition of 100 μL 4 × SDS loading buffer. For signaling analysis, the supernatants were removed at the indicated timepoints, and cells were washed once with PBS, after which cells were lysed with RIPA buffer. Electrophoresis was used to separate proteins in 8–15% polyacrylamide gels. After the proteins were transferred onto PVDF membranes, the blots were blocked with 5% skim milk. Primary antibodies were incubated overnight at 4°C, and secondary HRP-conjugated antibodies were incubated for 1 h at room temperature. Images were acquired using a GE Amersham Imager 600.

The following antibodies were used: anti-caspase-1 (AdipoGen, AG-20B-0042, 1:2,000), anti-caspase-3 (Cell Signaling Technologies [CST], #9662, 1:1,000), anti-cleaved caspase-3 (CST, #9661, 1:1,000), anti-caspase-7 (CST, #9492, 1:1,000), anti-cleaved caspase-7 (CST, #9491, 1:1,000), anti-caspase-8 (CST, #4927, 1:1,000), anti-cleaved caspase-8 (CST, #8592, 1:1,000), anti-ZBP1 (AdipoGen, AG-20B-0010-C100, 1:2,000), anti-NLRP3 (AdipoGen, AG-20B-0014, 1:2,000), anti-RIPK1 (CST, #3493, 1:1,000), anti-RIPK3 (CST, #95702, 1:1,000 or ProSci, #2283, 1:1,000), anti-pMLKL (CST, #37333, 1:1,000), anti-GFP (Santa Cruz Biotechnology, sc-8334, 1:1,000), anti-Flag (Sigma, F1804, 1:5,000), anti-GSDMD (Abcam, ab209845, 1:1,000), anti-GAPDH (CST, 5174, 1:5,000), anti-mCherry (Novus, 1-96752SS, 1:1,000), anti-FADD (Millipore, 05-486 1:1,000 or ENZO, ADI-AAM-212-E, 1:1,000), anti-ASC (AdipoGen, AG-25b-006-300, 1:1,000), and HRP-conjugated secondary antibodies (Jackson ImmunoResearch Laboratories, anti-rabbit [111-035-047], 1:5,000; anti-mouse [315-035-047], 1:5,000).

### Real-Time Cell Death Analysis

Real-time cell death analysis was performed as previously described (Malireddi et al., 2018). In brief, BMDMs were seeded in 24-well plates (0.5 × 10^6^ cells/well) and infected with *S*. Typhimurium, *L. monocytogenes*, VSV, or IAV or treated with LPS with or without TAK1i. Nuclei were stained using 20 nM SYTOX Green (Thermo Fisher Scientific, S7020). For VSV and IAV, SYTOX Green was added together with FBS following adsorption. Images were analyzed using IncuCyte S3 software. Masks used for quantification of the SYTOX Green stain are shown in red in the representative images.

### Immunoprecipitation

For the immunoprecipitation of NLRP3 and ZBP1, RIPK3, or RIPK1 in the overexpression system, HEK293T cells were seeded into six-well plates and transfected with 600 ng each of pcDNA3-N-FLAG-NLRP3 (Addgene, #75127), RIPK3-GFP (Addgene #41382), pCMV-mRIPK1, or pcDNA-mCherry-ZBP1 expression plasmids for 30 h. For the immunoprecipitation of the complex by RIPK3, HEK293T cells in a 10 cm dish were co-transfected with 600 ng each of pCMV6-mASC-turboGFP (Origene MG201872), pCDH-CMV-CASP8, pcDNA3-N-FLAG-NLRP3 (Addgene, #75127), RIPK3-GFP (Addgene #41382), pCMV-mRIPK1, and pcDNA-mCherry-ZBP1 expression plasmids for 30 h. The pan-caspase inhibitor zVAD-fmk was used to inhibit cell death due to the overexpression of these molecules. Subsequently, cells were lysed in NP-40 lysis buffer (1% NP-40, 150 mM NaCl, 50 mM HEPES, and protease inhibitor cocktail) for 20–30 min. Cell lysates were then centrifuged at 13,000 × g for 10 min. Supernatants were collected and incubated with 1–2 μg of the indicated antibody overnight at 4°C. Protein A/G-plus agarose beads (Santa Cruz sc-2003) were added into the lysates and incubated for 1–2 h at 4°C. After incubation, the beads were washed 4 times with lysis buffer and boiled in 2 × SDS loading buffer at 100°C for 5 min. Immunoprecipitates in sample buffer were subjected to immunoblotting analysis.

For immunoprecipitation in the endogenous system, fully differentiated primary wild-type C57BL/6 BMDMs were seeded 24 h prior to stimulations. BMDMs were stimulated with LPS (100 ng/mL) alone, LPS + TAK1-inhibitor (5Z-7-oxozeaenol [0.1 μM]) or LPS + TAK1-inhibior + zVAD (30 mM). After 4 h of stimulation, BMDMs were lysed in NP-40 lysis buffer. Whole cell lysates were harvested and incubated with 3 mg of indicated primary antibodies overnight at 4°C. Protein A/G plus agarose beads were added to these samples and incubated at 4°C for another 2 h. Agarose beads were centrifuged and washed in NP-40 lysis buffer three times, and immunoprecipitates were eluted by adding sample buffer. Immunoprecipitates in sample buffer were then subjected to immunoblotting analysis.

### Confocal Microscopy

Primary BMDMs fully differentiated from wild-type mice were infected with *S*. Typhimurium for 1.5 h at a multiplicity of infection of 1, fixed in 4% paraformaldehyde, and permeabilized in 0.5% Triton X-100 for 10 min. Samples were then blocked for 1 h in 5% bovine serum albumin in PBS. Samples were incubated with the following primary antibodies overnight at 4°C: anti-ASC (Millipore 04-147, diluted 1:100), anti-cleaved CASP3 (CST, #9661, diluted 1:250), and anti-phospho-MLKL (Santa Cruz, sc-165025, diluted 1:250). Samples were incubated with the following secondary antibodies for 1 h at room temperature: Alexa Fluor 488-conjugated antibody against mouse immunoglobulin G (Invitrogen, A21202, diluted 1:250), Alexa Fluor 568-conjugated antibody against rabbit immunoglobulin G (Invitrogen, A10042, diluted 1:250), and Alexa Fluor 647-conjugated antibody against goat immunoglobulin G (Invitrogen, A21447, diluted 1:250). Cells were counterstained with DAPI (Life Technologies), and confocal microscopy was performed using the Leica SP-8 confocal microscopy. Images were analyzed using FIJI software (ImageJ).

## Results

### Loss of PANoptotic Molecules Protects Against Cell Death After Infection

**Figure 1:**
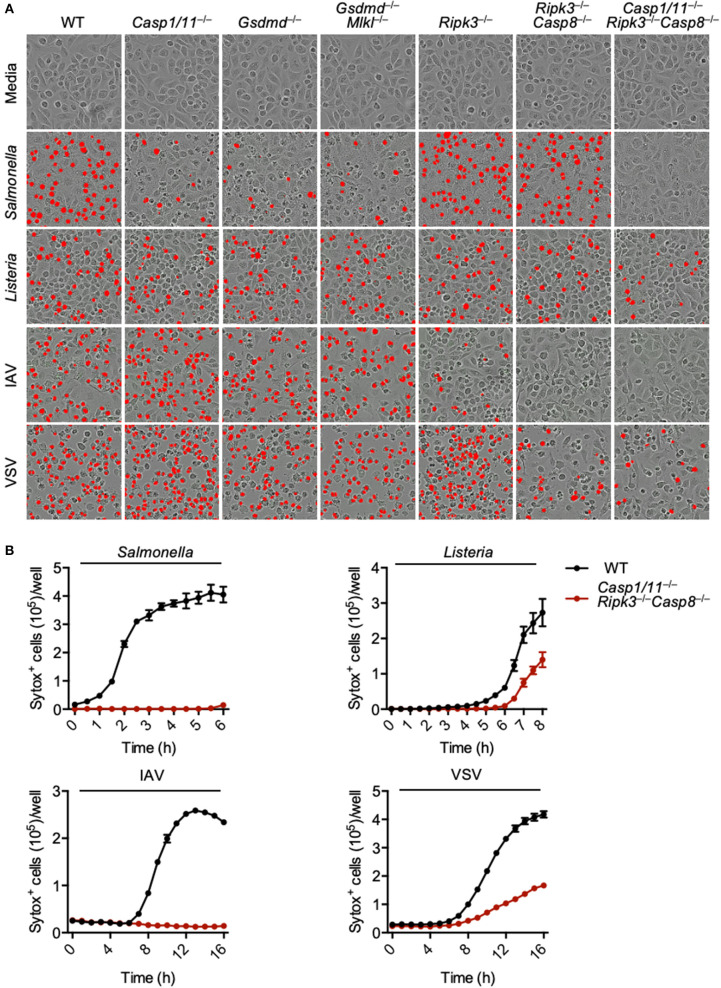
Loss of PANoptotic molecules prevents infection-induced cell death. Cell death analysis of BMDMs lacking different components of pyroptosis, apoptosis, or necroptosis. **(A)** Representative cell death images with the red mask indicating dead cells and **(B)** quantification of cell death over time in BMDMs after *S*. Typhimurium, *L. monocytogenes*, IAV, and VSV infection.

PANoptosis has been reported to occur after IAV infection and after loss of TAK1 activity (Kuriakose et al., 2016; Kesavardhana et al., 2017; Malireddi et al., 2018, 2020), but it is unclear what other pathogens can trigger this phenomenon. As necroptosis plays a role during viral infections and evidence suggests that necroptotic pathways become activated after *S*. Typhimurium or *L. monocytogenes* infection (Robinson et al., 2012; Jorgensen et al., 2017; Sai et al., 2019), we explored PANoptosis activation after *S*. Typhimurium, *L. monocytogenes*, IAV, and VSV infections. Infection of wild-type bone marrow-derived macrophages (BMDMs) with each of these pathogens resulted in robust cell death (Figure 1A). To determine the pathways involved in the induction of this cell death, we utilized a genetic approach and infected BMDMs derived from mice lacking key molecules involved in pyroptosis, apoptosis, and necroptosis. Pyroptosis was blocked by deletion of CASP1 and CASP11 or GSDMD; necroptosis was blocked by deletion of RIPK3 or MLKL; and apoptosis was blocked by deletion of CASP8. Loss of pyroptotic molecules (CASP1 and CASP11, or GSDMD) partially protected macrophages from *S*. Typhimurium-induced cell death, while loss of necroptotic (MLKL and RIPK3) and extrinsic apoptotic (CASP8) molecules had little impact. Cell death induced by *L. monocytogenes* was not robustly inhibited by loss of any single pathway or by the combined loss of pyroptosis and necroptosis or necroptosis and apoptosis. In the case of the viral infections, loss of necroptotic and apoptotic molecules partially protected against death. Loss of RIPK3 and CASP8 (necroptosis and extrinsic apoptosis) appeared to completely protect BMDMs from IAV-induced death. However, since IAV infection activates pyroptosis through the NLRP3 (nucleotide-binding oligomerization domain-like receptor [NLR] family pyrin domain-containing 3) inflammasome (Kanneganti et al., 2006a,b) and CASP8 has been shown to regulate NLRP3 inflammasome activation (Gurung et al., 2014), we have seen that pyroptosis is also blocked after this infection in the *Ripk3*^−/−^*Casp8*^−/−^ BMDMs (Zheng et al., 2020).

To eliminate the execution of all three pathways regulated by PANoptosis, we used BMDMs derived from mice lacking CASP1, CASP11, RIPK3, and CASP8. The *Casp1*/*11*^−/−^*Ripk3*^−/−^*Casp8*^−/−^ BMDMs were protected from *S*. Typhimurium or IAV-induced cell death at 6 and 16 h, respectively (Figures 1A,B). While loss of these components did reduce the death induced by *L. monocytogenes* and VSV infection, we still observed mild death in the *Casp1/11*^−/−^*Ripk3*^−/−^*Casp8*^−/−^ macrophages, suggesting the involvement of other pathways in addition to PANoptosis. Taken together, our findings suggest that PANoptosis is activated in response to these pathogens.

### Bacterial and Viral Infections Activate PANoptosis

**Figure 2:**
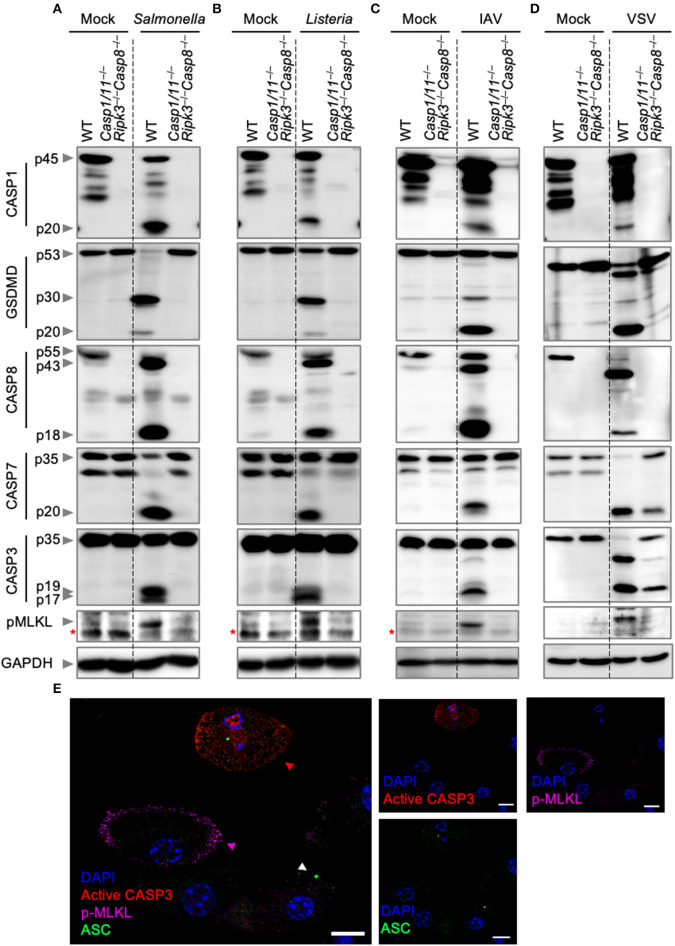
Bacterial and viral infections activate PANoptosis *in vitro*. Western blot analysis of PANoptosis activation markers after **(A)** *S*. Typhimurium, **(B)** *L. monocytogenes*, **(C)** IAV, and **(D)** VSV infection. Activation of pyroptosis was measured by immunoblotting of cleaved CASP1 (p20) and activated GSDMD (p20/p30). Activation of apoptosis was measured by immunoblotting of active CASP8 (p18), active CASP7 (p20), and active CASP3 (p17/19). Activation of necroptosis was measured by phosphorylation of MLKL (pMLKL). Red asterisks denote a non-specific band. **(E)** Confocal imaging of PANoptosis activation in wild-type BMDMs following *S*. Typhimurium infection. ASC speck formation (white triangle), cleavage of CASP3 (red triangle), and pMLKL localization to the membrane (purple triangle) were used as readouts of PANoptosis. Scale bar, 10 μm.

To further analyze the nature of cell death, we biochemically monitored the activation of PANoptosis by western blotting. Activation of pyroptosis was determined by CASP1 and GSDMD cleavage, while cleavage of CASP8, CASP3, and CASP7 were used as readouts for the activation of apoptosis. Necroptosis activation was monitored by the phosphorylation of MLKL. Consistent with our cell death assays, infection of wild-type BMDMs with *S*. Typhimurium, *L. monocytogenes*, IAV, or VSV led to activation of pyroptosis, apoptosis, and necroptosis (Figures 2A–D). These findings were further supported by visualization of the activation of the three pathways by confocal microscopy after *S*. Typhimurium infection (Figure 2E). Wild-type BMDMs infected with *S*. Typhimurium exhibited ASC speck formation (white triangle), indicative of the initiation of pyroptosis and consistent with the observed CASP1 activation. In addition, *S*. Typhimurium induced cleavage of CASP3 (red triangle), one of the effectors of apoptosis, and localization of phosphorylated MLKL to the cell membrane (purple triangle), consistent with necroptosis activation. Activation of these pathways was not observed in *Casp1*/*11*^−/−^*Ripk3*^−/−^*Casp8*^−/−^ BMDMs after *S*. Typhimurium or IAV infection. However, VSV infection of *Casp1*/*11*^−/−^*Ripk3*^−/−^*Casp8*^−/−^ BMDMs still resulted in the cleavage of CASP7 and CASP3 (Figure 2D). This is in line with our cell death data and consistent with previous studies demonstrating the activation of intrinsic (CASP9-mediated) apoptosis by VSV (Kopecky and Lyles, 2003; Felt et al., 2015). These findings suggest that intrinsic apoptosis contributes to cell death and is activated in addition to pyroptosis, extrinsic apoptosis, and necroptosis during VSV infection. We did not observe CASP3/7 cleavage in *Casp1*/*11*^−/−^*Ripk3*^−/−^*Casp8*^−/−^ BMDMs infected with *L. monocytogenes* (Figure 2B), suggesting that the residual death observed in these cells was not due to the activation of intrinsic apoptosis.

### Formation of the PANoptosome

**Figure 3:**
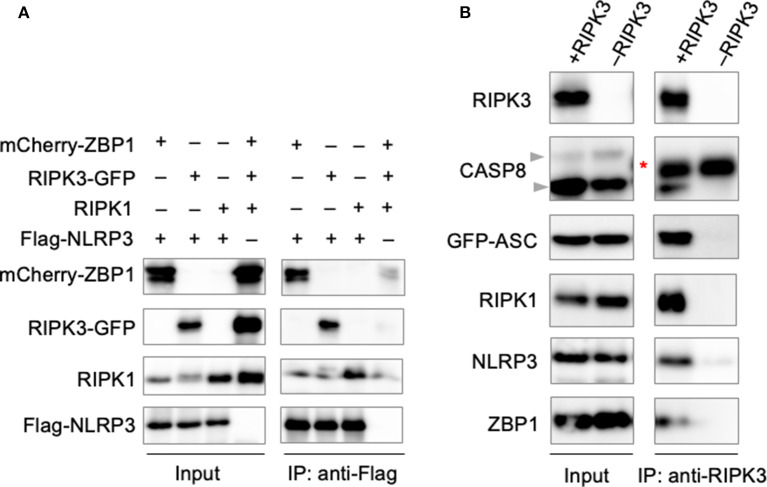
PANoptotic molecules directly interact. **(A)** Co-immunoprecipitation of NLRP3 expressed in HEK293T cells with ZBP1, RIPK3, and RIPK1 individually. **(B)** Co-immunoprecipitation of the PANoptosome complex in HEK293T cells expressing CASP8, ASC, RIPK1, NLRP3, and ZBP1 with or without RIPK3. Red asterisks denote a non-specific band.

The involvement of large signaling complexes in the induction of a number of programmed cell death pathways, along with previous findings demonstrating the colocalization of PANoptotic molecules (Malireddi et al., 2020), led us to explore the possibility that a PANoptosome complex comprised of pyroptotic, apoptotic, and necroptotic molecules forms to mediate PANoptosis. To interrogate the interactions between PANoptotic molecules, we transiently overexpressed molecules known to be essential to PANoptosis in HEK293T cells. Pulldown of FLAG-tagged NLRP3 resulted in the co-immunoprecipitation of ZBP1, RIPK3, and RIPK1 (Figure 3A), suggesting these molecules are capable of interaction. Furthermore, immunoprecipitation of RIPK3 from cells expressing components from all three pathways resulted in the co-immunoprecipitation of CASP8, ASC, RIPK1, NLRP3, and ZBP1 (Figure 3B), implying that these molecules may assemble into a single complex. Taken together, these data support the existence of direct interactions between PANoptotic molecules and the formation of a PANoptosome complex.

### Inhibition of TAK1 Results in PANoptosis Activation and Formation of the PANoptosome

**Figure 4:**
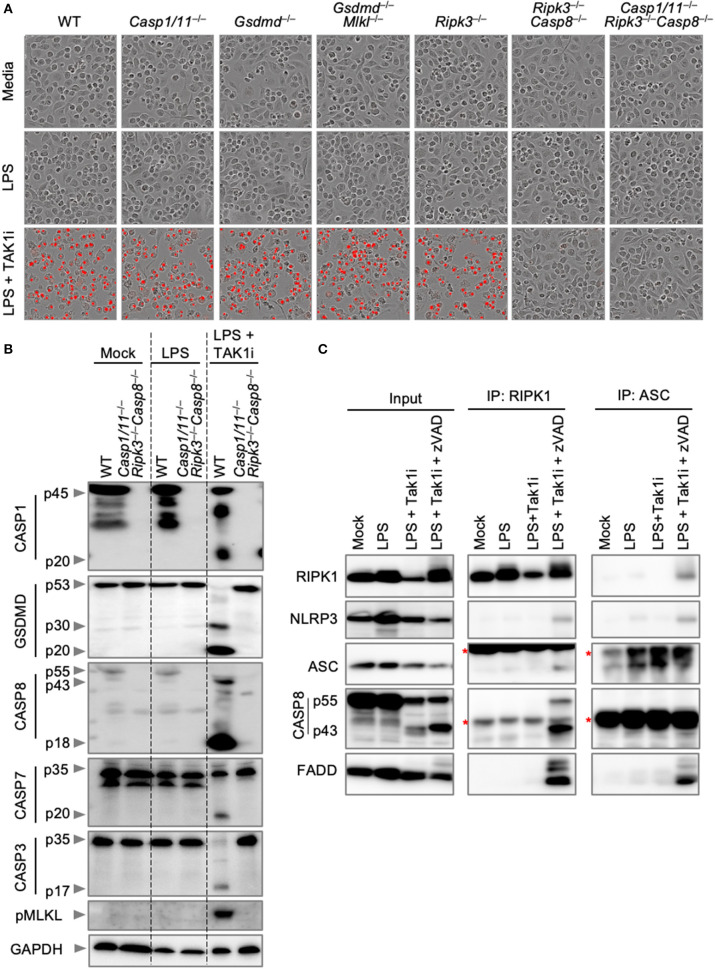
Inhibition of TAK1 promotes PANoptosis and PANoptosome formation. **(A)** Representative cell death images of BMDMs lacking different components of pyroptosis, apoptosis, or necroptosis after LPS priming and inhibition of TAK1. **(B)** Western blot analysis of PANoptosis activation after LPS priming and TAK1 inhibition. **(C)** Co-immunoprecipitation of PANoptosome components from primary BMDMs after TAK1 and caspase inhibition. Red asterisks denote a non-specific band.

We next sought to explore endogenous PANoptosome formation in response to a PANoptotic stimulus. TAK1 regulates cellular homeostasis, and loss of TAK1 results in the spontaneous activation of PANoptosis (Malireddi et al., 2018, 2020). In addition, the pathogenic *Yersinia* species inhibit TAK1 (Orning et al., 2018; Sarhan et al., 2018), and TAK1 inhibition during *Yersinia* infection leads to the activation of PANoptosis (Malireddi et al., 2018, 2020). TAK1 inhibition, therefore, provides a good model for the activation of PANoptosis in both sterile and infectious conditions. We first characterized cell death and PANoptosis activation in response to TAK1 inhibition. Treatment of BMDMs with an inhibitor of TAK1 (TAK1i, 5Z-7-Oxozeaenol) followed by LPS priming resulted in the induction of cell death in wild-type BMDMs (Figure 4A). Similar to the results observed with IAV infection, *Ripk3*^−/−^*Casp8*^−/−^ and *Casp1*/*11*^−/−^*Ripk3*^−/−^*Casp8*^−/−^ BMDMs were completely protected from cell death induced by TAK1i. This protection is due to the multifaceted roles of CASP8 in regulating both apoptosis and pyroptosis. Consistent with these findings and previous reports of PANoptosis activation following TAK1 inhibition, LPS priming of TAK1i-treated wild-type BMDMs induced activation of PANoptosis by immunoblot analysis (Figure 4B). These BMDMs displayed cleavage of CASP1, GSDMD, CASP8, CASP3, and CASP7 along with phosphorylation of MLKL, while LPS priming alone did not activate these pathways. However, there was minimal interaction observed between RIPK1 or ASC and other PANoptotic molecules from BMDMs treated with LPS and TAK1i (Figure 4C). As we have previously observed interactions between RIPK1 and NLRP3 after loss of TAK1 in the myeloid compartment, we hypothesized that the rapid cell death induction by TAK1i precluded capture of the intact PANoptosome complex. We therefore curbed the rapid proteolysis of the PANoptosome by treating BMDMs with the pan-caspase inhibitor z-VAD-fmk. Caspase inhibition in conjunction with LPS priming and TAK1 inhibition resulted in the co-immunoprecipitation of RIPK1, NLRP3, ASC, CASP8, and FADD when RIPK1 or ASC was immunoprecipitated (Figure 4C), suggesting that these molecules are all components of the PANoptosome complex induced by TAK1 inhibition and that loss of caspase activity helps allow the efficient recovery of this complex. Our findings here support that PANoptotic stimuli induce the formation of a PANoptosome complex comprised of a number of pyroptotic, apoptotic, and necroptotic molecules.

## Discussion

Recent studies have laid the foundation for our understanding of the extensive crosstalk between the inflammasome/pyroptosis and apoptosis and necroptosis (Lamkanfi et al., 2008; Malireddi et al., 2010, 2018, 2020; Gurung et al., 2014, 2016; Lukens et al., 2014; Kuriakose et al., 2016; Zheng et al., 2020), including the dual roles of apoptosis-associated FADD and CASP8 in mediating NLRP3 activation (Gurung et al., 2014), the involvement of TAK1 and RIPK1 in loss of cellular homeostasis and *Yersinia*-mediated pyroptosis (Malireddi et al., 2018, 2020; Orning et al., 2018; Sarhan et al., 2018), and the regulation of pyroptosis, necroptosis, and apoptosis by RIPK3 (Newton et al., 2014; Kuriakose et al., 2016; Nogusa et al., 2016; Zheng et al., 2020). PANoptosis is defined as a phenomenon whereby pyroptosis, apoptosis, and necroptosis become activated in the same cell population (Malireddi et al., 2019). In this study, we demonstrate that IAV, VSV, *S*. Typhimurium, and *L. monocytogenes* each activate PANoptosis. We further found that loss of the execution of the three PANoptotic pathways by genetic deletion of CASP1, CASP11, RIPK3, and CASP8 resulted in protection from the cell death induced by *S*. Typhimurium and IAV infection and partial protection from *L*. *monocytogenes* and VSV. Thus far, TAK1 and ZBP1 have been identified and demonstrated as master regulators of PANoptosis (Malireddi et al., 2019), though it is likely that additional undiscovered master regulators exist. In particular, our findings with *S*. Typhimurium infection, a condition that we have previously shown activates pyroptosis independently of ZBP1 or TAK1, suggest the involvement of at least one other unidentified regulator.

**Figure 5:**
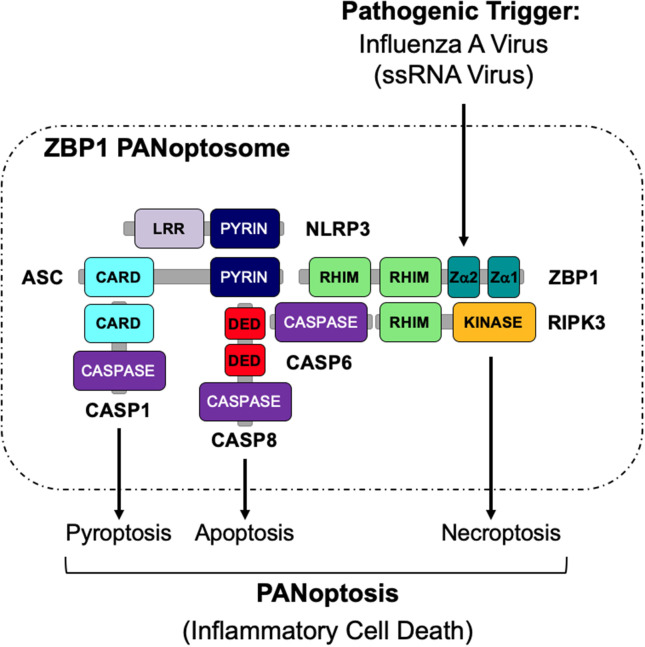
Graphical representation of a PANoptosome. Schematic summary of a representative PANoptosome formed after IAV infection. Domains hypothesized to be crucial for mediating PANoptosome formation are highlighted.

The activation of multiple cell death pathways through PANoptosis provides a way for host cells to prevent pathogens from evading detection. Previous studies suggest that the activation of these cell death pathways may be regulated by a single cell death complex that coordinates the interactions of key components of each pathway. Here, we show that RIPK1, RIPK3, CASP8, NLRP3, ASC, and FADD are capable of interacting to form a PANoptosome. The results presented here identify the assembly of a PANoptosome complex; however, the complete composition and the mechanisms governing the assembly of the PANoptosome are still unknown and warrant further investigation. Furthermore, it is likely that, similar to inflammasomes, the composition of a PANoptosome differs based on the PANoptotic stimulus and the innate immune sensor that recognizes that stimulus. As homotypic interactions are crucial to a number of signaling complexes, we propose that though individual PANoptosomes likely vary in composition, each PANoptosome will have components with key domains, including DEATH domains, DEDs, CARDs, PYRIN domains, and RHIMs that will facilitate assembly (Samir et al., 2020) (Figure 5). It is further possible that a defined set of proteins exists that are essential to promote the assembly of the PANoptosomes induced by different stimuli. For example, it was recently determined that caspase-6 (CASP6) promotes IAV-induced PANoptosis and plays a role in facilitating the interaction between ZBP1 and RIPK3 after IAV infection, implying that this protein is a component of the ZBP1 PANoptosome (Zheng et al., 2020). However, it is unclear if CASP6 plays a role in PANoptosis induced by other pathogens or stimuli.

Both VSV and *L. monocytogenes* induced cell death in the absence of CASP1/11, RIPK3, and CASP8. Cleavage of CASP3 and CASP7 in the *Casp1*/*11*^−/−^*Ripk3*^−/−^*Casp8*^−/−^ BMDMs during VSV infection suggests that this virus also activates intrinsic apoptosis, which has been previously reported (Kopecky and Lyles, 2003; Felt et al., 2015). We did not observe the same results with *L. monocytogenes*, suggesting the involvement of an unidentified mechanism of cell death. This residual cell death raises the question of whether PANoptosis and PANoptosomes also regulate other known pathways of programmed cell death in addition to pyroptosis, extrinsic apoptosis, and necroptosis, such as intrinsic apoptosis, or currently unknown and undescribed pathways of cell death. In depth dissection of all of the pathways regulated by the PANoptosome will be critical in future studies. Further research is also necessary to elucidate how components of the PANoptosome interact within a single complex and to determine the viability of the PANoptosome as a druggable target to modulate inflammatory cell death and aberrant immune responses.

## Data Availability Statement

All datasets presented in this study are included in the article.

## Ethics Statement

The animal study was reviewed and approved by St. Jude Children's Research Hospital committee on the Use and Care of Animals.

## Author Contributions

RK and T-DK conceptualized the study. SC, MZ, SK, and RK designed the methodology and conducted the analysis. SC, MZ, SK, RK, RM, BBr, DP, BBa, BS, ST, and PS performed the experiments. AB and BS generated the double knock out mouse line. SC and T-DK wrote the manuscript with input from all the authors. T-DK acquired the funding and provided overall supervision.

## Conflict of Interest

The authors declare that the research was conducted in the absence of any commercial or financial relationships that could be construed as a potential conflict of interest.
